# Facility-based care for moderately low birthweight infants in India, Malawi, and Tanzania

**DOI:** 10.1371/journal.pgph.0001789

**Published:** 2023-04-19

**Authors:** Katherine E. A. Semrau, Rana R. Mokhtar, Karim Manji, Shivaprasad S. Goudar, Tisungane Mvalo, Christopher R. Sudfeld, Melissa F. Young, Bethany A. Caruso, Christopher P. Duggan, Sarah S. Somji, Anne C. C. Lee, Mohamed Bakari, Kristina Lugangira, Rodrick Kisenge, Linda S. Adair, Irving F. Hoffman, Friday Saidi, Melda Phiri, Kingsly Msimuko, Fadire Nyirenda, Mallory Michalak, Sangappa M. Dhaded, Roopa M. Bellad, Sujata Misra, Sanghamitra Panda, Sunil S. Vernekar, Veena Herekar, Manjunath Sommannavar, Rashmita B. Nayak, S. Yogeshkumar, Saraswati Welling, Krysten North, Kiersten Israel-Ballard, Kimberly L. Mansen, Stephanie L. Martin, Katelyn Fleming, Katharine Miller, Arthur Pote, Lauren Spigel, Danielle E. Tuller, Linda Vesel, for the LIFE Study Group

**Affiliations:** 1 Ariadne Labs at Brigham and Women’s Hospital and the Harvard T.H. Chan School of Public Health, Boston, Massachusetts, United States of America; 2 Department of Medicine, Harvard Medical School, Boston, Massachusetts, United States of America; 3 Department of Pediatrics and Child Health, Muhimbili University of Health and Allied Sciences, Dar es Salaam, Tanzania; 4 Jawaharlal Nehru Medical College, KLE Academy of Higher Education and Research, Belgaum, Karnataka, India; 5 University of North Carolina Project Malawi, Lilongwe, Malawi; 6 Department of Pediatrics, School of Medicine, University of North Carolina at Chapel Hill, Chapel Hill, North Carolina, United States of America; 7 Department of Global Health and Population and Nutrition, Harvard T.H. Chan School of Public Health, Boston, Massachusetts, United States of America; 8 Hubert Department of Global Health, Rollins School of Public Health, Emory University, Atlanta, Georgia, United States of America; 9 Center for Nutrition, Division of Gastroenterology, Hepatology, and Nutrition, Boston Children’s Hospital, Boston, Massachusetts, United States of America; 10 Department of Nutrition, Harvard T.H. Chan School of Public Health, Boston, Massachusetts, United States of America; 11 Department of Pediatric Newborn Medicine, Brigham and Women’s Hospital, Boston, Massachusetts, United States of America; 12 Department of Nutrition, Gillings School of Global Public Health, University of North Carolina at Chapel Hill, Chapel Hill, North Carolina, United States of America; 13 Institute for Global Health and Infectious Diseases, School of Medicine, University of North Carolina at Chapel Hill, Chapel Hill, North Carolina, United States of America; 14 Department of Obstetrics and Gynecology, School of Medicine, University of North Carolina at Chapel Hill, Chapel Hill, North Carolina, United States of America; 15 Department of Obstetrics and Gynaecology, SCB Medical College and Hospital, Cuttack, Odisha, India; 16 Department of Obstetrics and Gynaecology, FM Medical College, Balasore, Odisha, India; 17 Department of Obstetrics and Gynaecology, City Hospital, Cuttack, Odisha, India; 18 Department of Paediatrics, SCB Medical College and Hospital, Cuttack, Odisha, India; 19 Department of Pediatrics, Harvard Medical School, Boston, Massachusetts, United States of America; 20 Maternal, Newborn, Child Health and Nutrition Program, PATH, Seattle, Washington, United States of America; Public Health Foundation of India, INDIA

## Abstract

Globally, increasing rates of facility-based childbirth enable early intervention for small vulnerable newborns. We describe health system-level inputs, current feeding, and discharge practices for moderately low birthweight (MLBW) infants (1500-<2500g) in resource-constrained settings. The Low Birthweight Infant Feeding Exploration study is a mixed methods observational study in 12 secondary- and tertiary-level facilities in India, Malawi, and Tanzania. We analyzed data from baseline facility assessments and a prospective cohort of 148 MLBW infants from birth to discharge. Anthropometric measuring equipment (e.g., head circumference tapes, length boards), key medications (e.g., surfactant, parenteral nutrition), milk expression tools, and human milk alternatives (e.g., donor milk, formula) were not universally available. MLBW infants were preterm appropriate-for-gestational age (38.5%), preterm large-for-gestational age (3.4%), preterm small-for-gestational age (SGA) (11.5%), and term SGA (46.6%). The median length of stay was 3.1 days (IQR: 1.5, 5.7); 32.4% of infants were NICU-admitted and 67.6% were separated from mothers at least once. Exclusive breastfeeding was high (93.2%). Generalized group lactation support was provided; 81.8% of mother-infant dyads received at least one session and 56.1% had 2+ sessions. At the time of discharge, 5.1% of infants weighed >10% less than their birthweight; 18.8% of infants were discharged with weights below facility-specific policy [1800g in India, 1500g in Malawi, and 2000g in Tanzania]. Based on descriptive analysis, we found constraints in health system inputs which have the potential to hinder high quality care for MLBW infants. Targeted LBW-specific lactation support, discharge at appropriate weight, and access to feeding alternatives would position MLBW for successful feeding and growth post-discharge.

## Introduction

Globally, nearly 20 million infants are born with low birthweight (LBW) (<2500g) each year. LBW infants represent a heterogeneous group who may be preterm (<37 weeks gestation) and/or small-for-gestational age (SGA; weight for gestation <10th percentile) [1]. LBW infants are at increased risk of morbidity, infections, growth deficits, developmental delays, and mortality [2].

The global increase in rates of facility-based childbirth (currently >75% of births) [3] enables greater opportunity to identify LBW and vulnerable infants near the time of birth to provide early, high quality, and appropriate care. Although 91% of LBW infants are born in low- and middle-income countries (LMIC) and the majority are moderately LBW (MLBW) (1500-<2500g) [4–6]; research is predominantly from high-income countries and focuses on very LBW (<1500g) or preterm infants [7]. MLBW infants are at higher risk for complications than normal weight (>2500g) infants and have been shown to be at greater risk of sepsis, temperature instability, and low blood sugar [8]. Currently, there is limited knowledge of facility-based feeding and discharge practices for MLBW infants, hindering proper management.

To help address these gaps, the Low Birthweight Infant Feeding Exploration (LIFE) study aimed to describe feeding and growth of MLBW infants in India, Malawi, and Tanzania [9]. In this analysis, we examined the health system inputs in the LIFE study facilities and described the current health facility care and feeding practices for MLBW infants from birth to discharge. The specific objectives were to: (1) examine the health system inputs available to support the care and feeding of MLBW infants; (2) understand the overall experience of facility care for MLBW infants with respect to the location and duration of their care and discharge practices; and (3) describe feeding practices from birth to facility discharge among MLBW infants.

## Methods

### Study design

LIFE is a formative, multi-site, observational cohort using a convergent parallel, mixed-methods design examining the feeding practices, growth, and health outcomes of MLBW infants (1500g≤ birthweight <2500g) in LMIC (Clinicaltrials.gov NCT04002908) [9]. Here, we present results from two quantitative data collection streams from LIFE: (i) a baseline facility needs assessment describing structural and service inputs of facilities where infants were enrolled, and (ii) a prospective in-facility observational cohort. Table 1 provides an overview of each data stream used in this analysis of the larger LIFE study **(Table 1)**.

**Table 1:** Overview of study objectives and data collection.

Data Stream	Facility needs assessment	In-facility observational cohort
**Aim**	To describe the health facility inputs as well as current facility care and feeding practices for MLBW infants from birth to discharge in LMICs in an effort to gather foundational knowledge and inform future interventions	
**Objectives**	*Health facility inputs* Examine the health system inputs (e.g., equipment, supplies, and human resources) available to support the care and feeding of MLBW infants	*Facility care practices* To understand the overall experience of facility care for MLBW infants with respect to the location and duration of their care and discharge practices *Facility feeding practices* To describe feeding practices from birth to facility discharge among MLBW infants
**Outcomes**	*Health facility inputs* • Facility level (secondary, tertiary) • Facility type (private/public) • Equipment • WASH supplies • Medication • Human resources	*Facility care practices* • Length of stay • Location of care • Separation between mother and infant • Weight at discharge • Adherence to documented facility discharge criteria • Feeding patterns at discharge *Facility feeding practices* • Provision of lactation support and management • Feeding profile • Early initiation of breastfeeding • Feeding competency
**Study design**	Observational, cross-sectional descriptive facility needs assessment prior to cohort enrollment	Formative research: observational, descriptive prospective cohort including direct observations and maternal reports
**Data collection and Study Population (N)**	Facility needs assessments: 12 health facilities (2–5 per site) India-Karnataka: 5 India-Odisha: 2 Malawi: 2 Tanzania: 3	In-facility observational cohort: 148 mother-infant pairs (35–40 per site) India-Karnataka: 38 India-Odisha: 35 Malawi: 35 Tanzania: 40
**Data analysis**	Descriptive statistics: means, medians, SD and frequencies	• Descriptive statistics: means, medians, SD and frequencies • Cochran-Mantel-Haenszel (CMH) and Chi-squared: p-values and confidence intervals for key indicators by LBW type, location of care and sex • Binomial regression: relative risk, 95% confidence interval, p-value

### Study facilities

The LIFE study was conducted in 12 health facilities in four sites across three countries (Dar-es-Salaam, Tanzania; Lilongwe, Malawi; Belgaum and Davangere, Karnataka State, India; and Cuttack, Odisha State, India) [9]. Each site had two to five study facilities chosen based on delivery volume, capacity to care for LBW infants, and willingness of facility leadership to participate. Study facilities included secondary and tertiary-level public-sector hospitals located in urban areas; in India-Karnataka, three private health facilities were also included **(Table 2**). The facility needs assessment was conducted between August-September 2019, prior to observational cohort enrollment.

**Table 2:** Facility-level resources for care of moderately low birthweight infants in 12 facilities in India, Malawi, and Tanzania.

Site		India-Karnataka (N = 5)					India-Odisha (N = 2)		Malawi (N = 2)		Tanzania N = 3)		
Facility details	Facility level	Tertiary	Tertiary	Tertiary	Tertiary	Tertiary	Secondary	Tertiary	Secondary	Tertiary	Secondary	Secondary	Tertiary
	Facility type	Private	Public	Private	Public	Private	Public	Public	Public	Public	Public	Public	Public
	Newborn care level*	III	II	III	II	III	II	II	II	III	I	I	III
	NICU beds	46	4–9	15	30	28	No NICU**	28	46	80	22	48	126
Human resources in NICU	Nurse to infant ratio												
	Day	1:3	1:6	1:4	1:7	1:9	No NICU	1:3	1:22	1:18	1:5	1:15	1:7
	Night	1:4	1:8	1:4	1:7	1:9	No NICU	1:7	1:33	1:35	1:5	1:25	1:10
	Physician to infant ratio												
	Day	1:3	1:10	1:4	1:6	1:6	No NICU	1:7	None	1:35	1:15	1:15	1:12
	Night	None	1:12	1:4	1:12	1:9	No NICU	1:35	None	None	1:20	1:20	1:25
Equipment for essential newborn care	Infant scale	✓	✓	✓	✓	✓	✓	✓		✓	✓	✓	✓
	Head circumference tape	✓	✓	✓	✓	✓		✓		✓ NICU only			✓
	Length board	✓	✓	✓ NICU only	✓	✓ NICU only		✓		✓ NICU only			
	Heat source for thermal care	✓	✓	✓	✓	✓	✓	✓	✓	✓	✓	✓	✓
	Bag and mask	✓	✓	✓	✓	✓	✓	✓	✓	✓	✓	✓	✓
	Continuous positive airway pressure (CPAP)	✓	✓	✓	✓	✓		✓	✓	✓	✓	✓	✓
	Ventilator	✓	✓	✓	✓	✓							✓
Equipment for feeding support	Total parenteral nutrition												✓
	Intravenous fluids (IV)	✓	✓	✓	✓	✓	✓	✓	✓	✓	✓	✓	✓
	Oral rehydration solution	✓	✓	✓	✓	✓	✓	✓	✓	✓	✓	✓	✓
	Donor human milk					✓							
	Preterm formula (any ward)		✓										✓
	Term formula (any ward)		✓						✓	✓	✓	✓	✓
	Manual breast pump							✓					
	Electric breast pumps					✓		✓					
	Designated location for milk expression	✓	✓	✓	✓			✓			✓		
	Alternative feeding supplies (cups/ spoons/ paladai)	✓	✓	✓	✓	✓	✓	✓	✓	✓	✓	✓	✓
	Nasogastric tube	✓	✓	✓	✓	✓	✓	✓	✓	✓		✓	✓
WASH for feeding supplies	Designated location for cleaning and prep	✓	✓	✓	✓	✓		✓	✓		✓	✓	✓
	Sink and treated water	✓	✓	✓	✓	✓		✓	✓	✓	✓		✓
Medication	Aminophylline, theophylline, or caffeine	✓	✓	✓	✓	✓		✓	✓	✓	✓	✓	✓
	Surfactant	✓	✓	✓									
	Intravenous dextrose	✓	✓	✓	✓	✓	✓	✓	✓	✓	✓	✓	✓

Check mark denotes present; blank cell denotes not present

*Newborn level of care definition based on guidance from AAP [11] and WHO/UNICEF Survive and Thrive [12]

**This facility does not have a formal NICU, but it has access to four warmers and basic resuscitation and oxygen facilities

### Study participants

After the needs assessment was completed in each facility, MLBW infants were eligible for the in-facility observational cohort if they were born or presented at one of the 12 study facilities during the enrollment period from August 2019 to April 2020. Each site enrolled between 35–40 infants in order to describe in detail the care of infants while in the facility. Our sample size was determined by considering timeline and budget constraints to conduct the intensive in-facility observation. Using a posthoc analysis with a sample size of 148 infants and 95% confidence, we were able to have a margin of error of 8% for common outcomes (50%) and 5% for an important feeding outcome of breastfeeding (90%). Infants were excluded if they had a birthweight <1500g or ≥2500g (n = 3); did not have maternal consent (n = 16); were born with congenital anomalies that could interfere with feeding (n = 3); were born to young mothers (n = 1) (i.e., <18 years in Tanzania and India; in Malawi, <16 years old if married or <17 years old if unmarried); or were born outside the facility (n = 10) [9]. Infants were also excluded if there was a maternal or newborn death, including stillbirth (n = 1) or death of a twin (n = 1).

### Ethics & consent

The LIFE study was approved by 11 ethics committees in India, Malawi, Tanzania, and the United States [9]. For the facility assessment, facility leadership gave verbal consent; for cohort enrollment, women provided written informed consent.

### Patient and public involvement

The LIFE study team involved clinicians, researchers, and community stakeholders who are familiar with the cultural context, study setting and populations in research design and study. In addition, study tools were piloted with mothers, community members, and health care providers as a way to capture culturally appropriate language, and ensure that surveys were acceptable.

### Data collection

#### Facility needs assessment

The facility assessment documented health facilities’ policies and capacity to provide care for LBW infants, with a focus on infant feeding and discharge practices. It assessed vital statistics (i.e. volume of births, volume of LBW births), structural inputs (i.e. infrastructure such as number of NICU beds, facility type, electricity, backup generators), human resources, and equipment available for MLBW care and feeding for each study facility [10, 11]. A study team member administered the assessment through direct observations, record/register reviews, and staff consultations, where needed. The level of care was defined based on recommendations from the American Academy of Pediatrics (AAP) (2012) [11], and guidance from the WHO/UNICEF Survive & Thrive report [12]. In our study, primary level facilities provide essential newborn care that includes immediate drying, skin to skin contact, early initiation and support of breastfeeding, outpatient care services and management and referral to higher level care. Secondary level facilities provide primary level care and address basic signal functions for small and sick newborns including the provision of extra warmth using incubators or radiant warmers in addition to Kangaroo Mother Care, administration of oxygen with continuous positive airway pressure (CPAP), resuscitation, detection and management of complications (such as neonatal encephalopathy (NEC), jaundice, infection, hypoglycemia) and access to a physician on call with specific neonatal skills in addition to specialized nurses/midwifery staff available at all times (24/7). Tertiary level facilities have a designated intensive care ward with secondary level capabilities as well as equipment for mechanical/assisted ventilation, specialized feeding equipment (e.g., total parenteral nutrition), nurses and doctors with specialized competencies in neonatal care available at all times (24/7), a neonatologist on call and access to other specialist doctors including pediatric surgery capabilities radiology, cardiology, neurology, and ophthalmology.

#### In-facility observational cohort

The in-facility observational cohort closely examined current infant feeding and discharge practices for MLBW infants. Mother-infant pairs were screened, consented, and enrolled within six hours of birth by trained study personnel and followed during daylight hours (7:00am to 5:00pm, daily in India and Tanzania; on weekdays in Malawi) until facility discharge. Demographics, birth characteristics, infant feeding patterns, infant feeding competency, and neonatal weight changes were documented from birth until facility discharge.

Demographic information was extracted from medical records. Gestational age (GA) was based on ultrasound, last menstrual period (LMP), or fundal height recorded in a patient’s chart with priority given to first trimester ultrasound followed by documented LMP. Using the WHO growth standards for term infants [13, 14] and the INTERGROWTH-21st growth standards for preterm infants [15], infant LBW type were identified: preterm-small for gestational age (preterm-SGA), preterm-appropriate for gestational age (preterm-AGA), preterm-large for gestational age (preterm-LGA), and term-SGA [1]. Mother-infant separation, based on maternal report, was defined as the time the mother and infant were not sharing a room.

Trained study nurses directly observed feeding sessions and interviewed mothers about practices between observations regarding feeding patterns (e.g., direct breastmilk, expressed breastmilk, donor human milk (DHM), animal milk, formula, or another liquid), and lactation support (e.g., verbal advice, physical support, timing) at each visit. Observation started within six hours after birth and occurred every three hours for the first seven days, twice daily for days 8–14, and then once a day for unstable infants or every three days for stable infants until discharge. Both direct observation and maternal report were used to reduce the possibility of bias and allow for triangulation of data. The Preterm Infant Breastfeeding Behavior Scale (PIBBS), a 9-question validated tool, assessed feeding competency based on direct feeding observation by trained study nurses measuring infant rooting efforts, latch effort, latch duration, sucking duration, and swallowing; scores <15 (out of 20) signified poor feeding competency [16]. Timing of breastfeeding initiation was collected at the baseline observational visit within the first six hours after birth. Early initiation of breastfeeding, a validated Infant and Young Child Feeding indicators for feeding practices [17], was calculated as the frequency of infants who were breastfed within less than 1 hour.

Birthweight measured by clinical staff was used to assess MLBW infant study eligibility. After study enrollment and within six hours of birth, trained study staff measured study specific birthweight and other infant anthropometrics, in triplicate, using standardized and calibrated study specific equipment at birth. Measurements were repeated just prior to discharge [9]. The average of the two (out of the three) closest measurements was used for analysis. Birth and discharge weight were used to calculate absolute and proportional weight change [18, 19].

### Data analysis

Descriptive statistics included mean, standard deviation (SD), median and interquartile range (IQR) for continuous variables, and frequencies and percentages for categorical variables. Chi-squared test was used to determine statistical differences between preterm birth and geographical region. Cochran-Mantel-Haenszel (CMH) chi-square test was used to compare differences of lactation support and counseling by sex, LBW type, and NICU admission after controlling for study site. We analyzed data by location of care (NICU or postpartum ward) [20]. A one-way analysis of variance was performed with a Bonferroni correction to compare differences in the mean gestational age by LBW type. Wilcoxon rank sum test was used to determine the difference between median birthweight by site, median length of stay differences by location of care (NICU vs postpartum ward), sex and LBW type. Crude binomial regression models, adjusted for clustering by health facility with compound symmetry and a log link function, were used to estimate relative risk ratios (RR) for the relationship between admission to the NICU and LBW type or birthweight bands (1500-<1800g, 1800-<2200g, 2200-<2500g) as well as the association between early initiation of breastfeeding (<1 hour) and mother-infant separation. Due to the small sample size, intended mainly for descriptive analyses, we did not adjust for any potential confounders in our analyses and only adjusted for clustering by facility. Exploration of the data demonstrated that missing data was rare; however, data were likely missing randomly or due to data collection difficulties; we excluded missing data from analysis. Data were analyzed using SAS 9.3 (SAS Institute. SAS Software Suite. Cary, NC: SAS Institute; 2020) and Microsoft Excel.

## Results

### Facility characteristics

The facility assessment was completed prior to the in-facility observational cohort enrollment to understand currently available facility resources, demonstrating variation in access to general equipment, medications, infant feeding supplies, and level of care across the facilities (**Table 2**). Weighing scales were mostly available, but length assessment tools were not.

All 12 facilities were accredited by the Baby-Friendly Hospital Initiative (BFHI) and prioritized breastfeeding [21]. All had supplies to support infant feeding including paladais and cups/spoons; most facilities (11/12) had nasogastric tubes. Infant formula was only used when a mother was absent or unable to express breastmilk. Half of the facilities had powdered infant formula in stock; two facilities had preterm formula; and none had pre-mixed liquid formula. Reportedly, formula was often purchased by families if required for their infant.

All 12 facilities followed formalized discharge policies based on national guidelines; 9/12 had facility-specific guidelines. At the time of discharge in all facilities, infants had to be feeding adequately, hemodynamically stable, and able to consume breastmilk (directly or expressed). Although not the sole criteria, policy-stated minimum discharge weight criteria varied by site: 2000g in Tanzania, 1800g in India, and 1500g in Malawi.

### Characteristics of the in-facility observational cohort

We screened 183 MLBW infants and enrolled 148 (80.9%) **(Table 3);** 1076 observations were completed among the 148 infants during their facility admission. Three infants in Malawi died prior to facility discharge. Prematurity was more common in the African sites (62.0% in Tanzania and 62.9% in Malawi) compared to the Indian sites (36.8% in India-Karnataka and 31.4% in India-Odisha) (p<0.0001). The mean GA was 35.6 weeks (0.9) for preterm-SGA, 33.5 weeks (1.7) for preterm-AGA, 30.1 weeks (1.7) for preterm-LGA, and 38.9 weeks (1.7) for term-SGA infants (p<0.0001). The median birthweight [2145g (1924, 2280] did not vary by site (p = 0.14) **(Table 3)**.

**Table 3:** Maternal and infant characteristics in a cohort of 148 moderately low birthweight infants in 12 facilities in India, Malawi, and Tanzania.

Indicator of interest		India-Karnataka	India-Odisha	Malawi	Tanzania	Pooled
**Maternal**		**N = 37**	**N = 35**	**N = 35**	**N = 35**	**N = 142**
Maternal age (years)	Mean (SD), range	23.7 (3.7), 19.0–35.0	25.6 (5.4), 19.0–38.0	28.1 (7.2), 17.0–40.0	29.0 (6.3), 19.0–43.0	26.5 (6.1), 17.0–43.0
Marital status n (%)	Married	37 (100.0%)	34 (97.4%)	32 (91.4%)	28 (80.0%)	131 (92.3%)
Maternal education n (%)	Primary or less	12 (32.4%)	10 (28.6%)	22 (62.9%)	19 (54.3%)	63 (44.4%)
	Secondary or more	25 (67.6%)	25 (71.4%)	13 (37.1%)	16 (45.7%)	79 (55.6%)
Paternal education n (%)	Primary or less	12 (32.4%)	7 (20.0%)	17 (48.6%)	17 (48.6%)	53 (37.3%)
	Secondary or more	25 (67.6%)	26 (74.3%)	15 (42.8%)	16 (45.7%)	82 (57.7%)
	Not available (widowed/ divorced/single)	0 (0%)	2 (5.7%)	3 (8.6%)	2 (5.7%)	7 (5.0%)
Maternal religion n (%)	Hindu	31 (83.8%)	34 (97.1%)	N/A	N/A	65 (45.8%)
	Muslim	6 (16.2%)	1 (2.9%)	2 (5.7%)	25 (71.4%)	34 (23.9%)
	Christian*	N/A	N/A	28 (80.0%)	10 (28.6%)	38 (26.8%)
	No religion	N/A	N/A	1 (2.9%)	0 (0%)	1 (0.7%)
	Other	N/A	N/A	4 (11.4%)	0 (0%)	4 (2.8%)
Place of residence n (%)	Rural	24 (64.9%)	24 (68.6%)	14 (40.0%)	0 (0%)	62 (43.7%)
	Urban	13 (35.1%)	11 (31.4%)	21 (60.0%)	35 (100%)	80 (56.3%)
ANC attendance n (%)	Yes	35 (94.6%)	30 (85.7%)	34 (97.1%)	35 (100%)	134 (94.4%)
Mother’s parity n (%)	1	16 (43.2%)	24 (68.6%)	9 (25.7%)	12 (34.3%)	61 (43.0%)
	2–4	21 (56.8%)	11 (31.4%)	19 (54.3%)	23 (65.7%)	74 (52.1%)
	5+	0 (0%)	0 (0%)	7 (20.0%)	0 (0%)	7 (4.9%)
Number of babies born in this delivery n (%)	Singleton	35 (94.6%)	35 (100%)	32 (91.4%)	28 (80.0%)	130 (91.6%)
	Twins**	2 (5.4%)	0 (0%)	3 (8.6%)	7 (20.0%)	12 (8.5%)
Mother living with HIV n (%)	Yes	0 (0%)	0 (0%)	5 (14.3%)	5 (14.3%)	10 (7.0%)
Maternal complications at birth as noted in patient chart n (%)	Normal (none)	29 (78.4%)	33 (94.3%)	21 (60.0%)	25 (71.4%)	108 (76.1%)
	Fever/infection	2 (5.4%)	0 (0%)	0 (0%)	1 (2.9%)	3 (2.1%)
	Heavy/excessive bleeding	0 (0%)	0 (0%)	1 (2.9%)	0 (0%)	1 (0.7%)
	High blood pressure/ preeclampsia	4 (10.8%)	1 (2.9%)	7 (20%)	9 (25.7%)	20 (14.1%)
	Convulsions	0 (0%)	0 (0%)	1 (2.9%)	0 (0%)	1 (0.7%)
	Other	4 (10.8%)	2 (5.7%)	6 (17.1%)	0 (0%)	12 (8.5%)
	No data in chart	0 (0%)	0 (0%)	1 (2.9%)	0 (0%)	1 (0.70%)
**Infant**		**N = 38**	**N = 35**	**N = 35**	**N = 40**	**N = 148**
Infant’s sex n (%)	Male	15 (39.5%)	16 (45.7%)	13 (37.1%)	19 (47.5%)	63 (42.6%)
	Female	23 (60.5%)	19 (54.3%)	22 (62.9%)	21 (52.5%)	85 (57.4%)
Mean birthweight (grams)	Mean (SD), range	2150 (244), 1590–2485	2115 (218), 1575–2480	2008 (300), 1400–2460	2081 (241), 1520–2490	2089 (255), 1400–2490
Median birthweight (grams)	Median (IQR)	2250 (1975, 2345)	2160 (1950, 2270)	2105 (1700, 2210)	2105 (1913, 2285)	2145 (1924, 2280)
Birthweight categories n (%)	1500 - <1800 g	4 (10.5%)	3 (8.6%)	10 (28.6%)	5 (12.5%)	22 (14.9%)
	1800 - <2200 g	11 (29.0%)	18 (51.4%)	15 (42.8%)	19 (47.5%)	63 (42.6%)
	2200 - <2500 g	23 (60.5%)	14 (40.0%)	10 (28.6%)	16 (40.0%)	63 (42.6%)
Mean gestational age at birth	Mean (SD)	37.1 (2.7)	37.5 (2.6)	34.1 (3.1),	36.0 (3.2)	36.2 (3.1)
Infant LBW type n (%)	Preterm-SGA	3 (7.9%)	6 (17.1%)	4 (11.4%)	4 (10.0%)	17 (11.5%)
	Preterm-AGA	11 (28.9%)	4 (11.4%)	18 (51.4%)	24 (60.0%)	57 (38.5%)
	Preterm-LGA	0 (0%)	1 (2.9%)	4 (11.4%)	0 (0%)	5 (3.4%)
	Term-SGA	24 (63.2%)	24 (68.6%)	9 (25.7%)	12 (30.0%)	69 (46.6%)
Delivery mode n (%)	Vaginal delivery	25 (65.8%)	31 (88.6%)	29 (82.9%)	31 (77.5%)	116 (78.4%)
	Cesarean delivery	12 (31.6%)	4 (11.4%)	6 (17.1%)	9 (22.5%)	31 (20.9%)
	No data in chart	1 (2.6%)	0 (0%)	0 (0%)	0 (0%)	1 (0.7%)
Length of stay in facility (days)	Median (IQR), range	5.1 (3.2, 7.0), 0.0–27.2	2.0 (1.3, 2.3), 1.1–19.2	2.3 (1.2, 6.0), 1.0–30.5	3.8 (2.1, 5.2), 1.0–16.5	3.1 (1.5, 5.7), 0.0–30.5
Place discharged n (%)	Discharged to home	36 (94.7%)	35 (100%)	32 (91.4%)	39 (97.5%)	142 (95.9%)
	Referred	0 (0%)	0 (0%)	0 (0%)	1 (2.5%)	1 (0.7%)
	Left against medical advice	1 (2.6%)	0 (0%)	0 (0%)	0 (0%)	1 (0.7%)
	Not applicable, died	0 (0%)	0 (0%)	3 (8.6%)	0 (0%)	3 (2.0%)
	Missing	1 (2.6%)	0 (0%)	0 (0%)	0 (0%)	1 (0.7%)

* Includes Catholic, Anglican, Protestant, Seventh Day Advent/Baptist, other Christian

** Twins were eligible for enrollment; at times, only one infant of the twin pair was enrolled. The breakdown of twin enrollment by site was: Tanzania: two pairs of twin’s enrolled only one twin; Malawi: three pairs of twin’s enrolled only one twin; India-Karnataka: one pair of twins enrolled only one twin; and India-Odisha: no twins were enrolled

The median length of stay, which varied by site (p = 0.01), was 3.1 days [IQR: 1.5, 5.7]; 75% of infants were discharged within 7 days (**Table 3**). Length of stay also varied by infant LBW type: preterm-AGA infants [median (IQR): 4.4 days (2.0, 7.3)] stayed longer than preterm-SGA [2.4 days (2.0, 4.5)], preterm-LGA [1.3 days (1.1, 1.9)], and term-SGA [2.3 days (1.5, 4.3)] infants (p = 0.02). Infants born in secondary-level facilities were discharged earlier than those in tertiary facilities.

### Location of MLBW infant care

#### Separation

Based on maternal report, 67.6% (100/148) infants were separated from their mothers during their time in the facility. Occurrence of separation significantly varied across sites, occurring most often in the African sites [Malawi: 82.9% (29/35); Tanzania: 95.0% (38/40)] compared to the Indian sites [India-Karnataka: 39.5% (15/38); India-Odisha: 51.4% (18/35)]. Out of 100 mother-infant dyads separated, 52 (52.0%) were separated within 30 minutes postpartum and the vast majority were separated within 6 hours postpartum (93/100). Mothers reported the predominant reasons for separation were due to policies related to premature birth (50.0%), LBW (80.0%), facility standard practice (20.0%), infant illness (21.0%), or maternal illness (13.0%). Across the duration of admission, separation lasted an average of 21.0 hours (IQR: 4.0, 72.0) and did not vary by sex, but varied significantly by site (p<0.0001), with the longest in Tanzania and shortest in India-Odisha [median (IQR) hours: India-Karnataka: 9.0 (3.0, 36.0); India-Odisha: 2.0 (1.0, 7.0); Malawi: 5.0 (2.0, 18.0); Tanzania: 55.0 (28.0, 100.0)]. Term-SGA infants had the shortest duration of separation, but this was not significant [(preterm-SGA (n = 8): 17.5 (2.0, 61.0); preterm-AGA (n = 47): 25.0 (5.0, 75.0); preterm-LGA (n = 3): 2.0 (1.0, 27.0), term-SGA (n = 28): 6.5 (2.0, 44.0) (p = 0.69)]. Percent of time separated, calculated by dividing the total separation in hours by total hours of facility stay, demonstrated that mother-infant dyads spent more than one-third of their admission separated (37.3% (SD: 37.6%), n = 86).

#### NICU

Over the entire length of stay, 32.4% (48/148) of infants were admitted to the NICU, primarily in tertiary facilities (87.5%, 42/48). NICU admission was most common in Tanzania (42.5%, 17/40) and Malawi (42.9%, 15/35), followed by India-Karnataka (37.8%, 14/35) and India-Odisha (5.7%, 2/35), depending on policy standards and facility availability. Admission to the NICU was most prevalent among preterm-AGA infants (50.9%) followed by term-SGA (21.7%), preterm-LGA (20.0%) and preterm-SGA infants (17.7%). In crude binomial regression models adjusting for clustering by facility, there was no significant association between preterm-SGA and term-SGA infants and NICU admission compared to preterm-AGA infants [(RR: 0.61, 95% CI: 0.32–1.19, p = 0.15) and (RR: 0.68, 95% CI: 0.31–1.5, p = 0.34), respectively]; conversely, preterm-LGA infants were significantly less likely to be admitted to the NICU compared to preterm-AGA infants [RR: 0.38, 95% CI: 0.23–0.62, p<0.0001]. In addition, infants with the lowest birthweights [(1500-<1800g) and (1800 -<2200g)] had a higher prevalence of admission to the NICU (54.6% and 31.8%) compared to those infants born with higher birthweights bands (2200-<2500g: 25.4%). However, this was not significant after adjusting for clustering by facility [(RR (1500-<1800g): 1.3, 95% CI: 0.61–3.0, p = 0.47) and (RR (1800-<2200g: 1.2, 95% CI: 0.76–1.8, p = 0.45)]. Infants ever admitted to the NICU had a significantly longer length of stay compared to non-NICU admitted infants. Infants admitted to the NICU had a median length of stay of 6.0 days (IQR: 4.3, 7.7) compared with 2.1 days (IQR: 1.3, 3.3) among infants in the postpartum ward (p<0.0001). In addition, the length of stay varied significantly by site (p<0.0001). NICU admission duration did not vary by infant sex or LBW type.

Infants in the NICU were primarily fed breastmilk unless clinical complications impacted the infants’ ability to feed. Oxygen/CPAP was initiated for 43.8% (21/48) of NICU admitted infants. Feeding intolerance occurred among 22.9% (11/48) of the NICU-admitted infants, which led clinicians to stop oral feeds. These infants were then given parenteral nutrition, fed breastmilk via nasogastric tube, and/or were administered intravenous fluids. Of all the NICU-admitted infants, 4.2% (2/48) were given parenteral nutrition, 8.3% (4/48) had a nasogastric tube, and 54.2% (26/48) were given fluids intravenously.

### Feeding from birth to facility discharge

#### Practices

**Fig 1:**
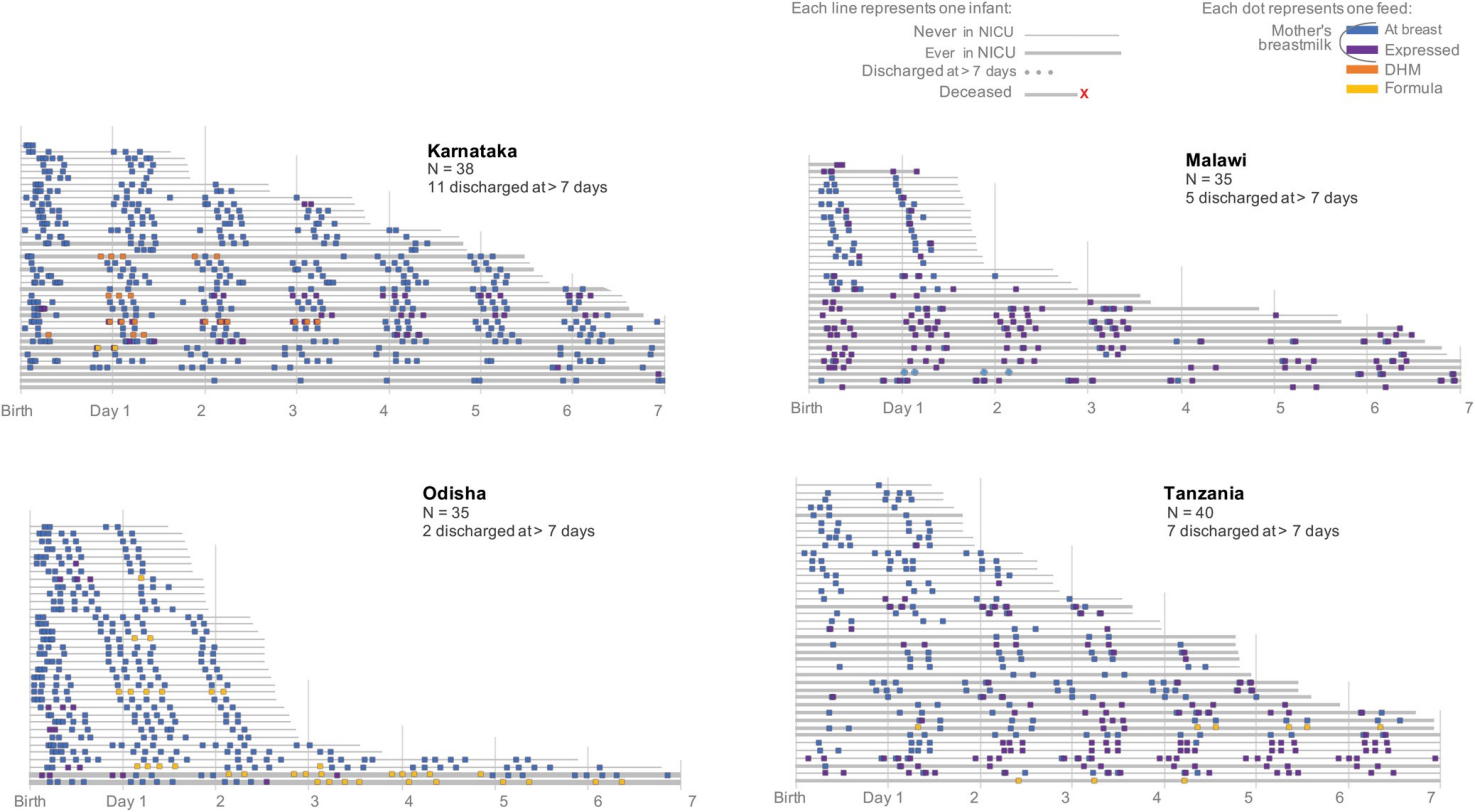
Observed feeding patterns in the first week of life prior to facility discharge among moderately low birthweight infants across 12 facilities in India, Malawi, and Tanzania.

Of the 148 enrolled infants, one infant in Malawi did not have any feeds prior to discharge at two days postpartum; the remaining 147 infants had feeding observed from birth to discharge. The majority, 93.2% (137/147), were exclusively fed human milk [mom’s own milk or DHM], 6.1% (9/147) were given mixed milk feeding [formula and breastmilk], 2.0% (3/147) were given other liquids (no specific information available), and 0.7% (1/147) was given only formula. Mixed milk feeding was more common in India-Odisha (6.1%, 6/35) compared to Tanzania (2.5%, 1/40), Malawi (2.9%, 1/34), and India-Karnataka (2.6%, 1/40). Provision of feeds other than human milk and formula (e.g., provision of other foods, teas, or liquids) were not observed. Based on maternal report, three infants were given other unknown liquids. Otherwise, feeding information (direct breastfeeding, milk expression, and/or infant formula use), obtained from direct observations was highly correlated with maternal self-report (**Fig 1**).

Of the 82 mothers with available feeding initiation data, 81.7% (67/82) initiated breastfeeding within 1 hour of birth. Early initiation varied by infant LBW type: 90.0% of preterm-SGA, 59.1% of preterm-AGA, 25.0% of preterm-LGA, and 95.7% of term-SGA infants. This variation was likely related to mother-infant separation and infant prematurity with preterm-AGA and preterm-LGA infants averaging 33.5 and 30.1 weeks gestation respectively, preterm-SGA infants at 35.6 weeks and term-SGA at 38.9 weeks. Among infants separated, 69.0% (29/42) initiated early breastfeeding, whereas 95% (38/40) of non-separated infants initiated early breastfeeding. After adjusting for clustering by facility in a crude binomial regression model, infants who were separated from their mothers were significantly less likely to initiate early breastfeeding compared to those infants who were not separated [RR: 0.26, 95% CI: 0.10–0.42, p = 0.0013].

Direct breastfeeding was the predominant feeding type observed (94.6%, 139/147) (**Figs 1 and S1**) followed by expressed breastmilk (43.5%, 64/147). Provision of expressed breastmilk, unfortified and fed immediately after expression, varied by site (expression uptake by site: Tanzania: 55.0%; Malawi: 70.6%; India-Karnataka: 29.0%; India-Odisha: 20.0%; p<0.001). Direct observation of milk expression was conducted with 62/64 mothers. Nearly all expressed milk by hand (98.4%, 61/62); a manual pump was used by three mothers (4.8%, 3/62), and electric pumps by two (3.2%, 2/62) at private facilities.

Formula was fed to 6.1% (9/147) of infants with a cup/spoon/paladai, primarily in India-Odisha and Tanzania; bottles with a nipple were not used. Unfortified DHM fed by cup/spoon/paladai was given to 10.5% (4/38) of infants born in India-Karnataka, representing 2.7% of the whole cohort. Supplementation, only given in India-Karnataka, included iron and vitamin D (56.8%, 21/37) or zinc and multivitamins (24.3%, 9/37).

#### Lactation support and counseling

Overall, 81.8% of mothers reported receiving at least one lactation support and counseling session; yet, the frequency, provider, and type of counseling varied by site (**Table 4**). Two or more sessions were provided to 56.1% of dyads, typically in a group setting. Support was primarily provided by healthcare providers (90.1%) or family members (27.3%). Type of support varied by site, including help with positioning of the infant, latch, milk expression, and feeding with a cup/paladai (**Table 4**). Counseling, as reported by mothers, did not vary by infant sex (χ^2^_cmh_ = 2.85, p = 0.09), LBW type (χ^2^_cmh_ = 0.004, p = 0.95), or NICU admission (χ^2^_cmh_ = 0.87, p = 0.35). Mothers reported being the primary decision-makers for breastmilk feeding. Clinicians played an important role in influencing the mother to feed infants expressed breastmilk (68.1%), DHM (100.0%), and infant formula (100.0%). For the three infants whose mothers reported to feed ‘other’ unknown liquids, the mothers (n = 2) or family members (n = 1) made the decision.

**Table 4:** Feeding support and counseling received in the facility based on maternal report and observations in a cohort of 148 moderately low birthweight infants in 12 facilities in India, Malawi, and Tanzania.

Feeding support and counseling indicator	India-Karnataka	India-Odisha	Malawi	Tanzania	Pooled
Ever received feeding support (during time in the facility)	N = 38	N = 35	N = 35	N = 40	N = 148
Ever received based on maternal report	36 (94.7%)	23 (65.7%)	29 (82.9%)	33 (82.5%)	121 (81.8%)
Ever received two or more support sessions based on maternal report	29 (76.3%)	9 (25.7%)	24 (68.6%)	21 (52.5%)	83 (56.1%)
Ever received based on observation	32 (84.2%)	19 (54.3%)	26 (74.3%)	27 (67.5%)	104 (70.3%)
Median (IQR) number of times mother reported receiving feeding support at facility within first 0–7 days of life (n = 146)	3.5 (2.0, 7.0)	1.0 (0, 2.0)	2 (1.0, 3.0)	1.5 (1.0, 3.5)	2 (1.0, 4.0)
Median (IQR) number of times mother reported receiving feeding support at facility >7 days—3 weeks of life (n = 23)	13.0 (7.0, 9.0)	4.5 (0, 9.0)	4.0(2.0, 6.0)	5.0 (2.0,11.0)	7.0 (2.0, 13.0)
MATERNAL REPORT: Types of support received*	**N = 36**	**N = 23**	**N = 29**	**N = 33**	**N = 121**
Talking about proper latch/positioning	31 (86.1%)	20 (87.0%)	12 (41.4%)	30 (90.9%)	93 (76.9%)
Support with positioning mom/baby	31 (86.1%)	8 (34.8%)	11 (37.9%)	16 (48.5%)	66 (54.6%)
Support with latching	16 (44.4%)	3 (13.0%)	10 (34.5%)	8 (24.2%)	37 (30.6%)
Support with expressing breastmilk	7 (19.4%)	3 (13.0%)	25 (86.2%)	13 (39.4%)	48 (39.7%)
Support for feeding with bottle/cup/paladai	4 (11.1%)	4 (17.4%)	22 (75.9%)	9 (27.3%)	39 (32.2%)
Other	4 (11.1%)	0	15 (51.7%)	0	19 (15.7%)
**MATERNAL REPORT: Provider of support**	**N = 36**	**N = 23**	**N = 29**	**N = 33**	**N = 121**
Health care provider**	34 (94.4%)	13 (56.5%)	29 (100.0%)	33 (100.0%)	109 (90.1%)
Lactation consultant	2 (5.6%)	0	0	0	2 (1.7%)
Community health worker	2 (5.6%)	3 (13.0%)	0	0	5 (4.1%)
Family member	20 (55.6%)	12 (52.2%)	1 (3.5%)	0	33 (27.3%)
Friend	2 (5.6%)	0	0	1 (3.0%)	3 (2.5%)
Other	1 (2.8%)	0	0	0	1 (0.83%)
**OBSERVATION: Types of support received**	**N = 32**	**N = 19**	**N = 26**	**N = 27**	**N = 104**
Talking about proper latch/positioning	28 (87.5%)	17 (89.5%)	10 (38.5%)	24 (88.9%)	79 (76.0%)
Support with positioning mom/baby	30 (93.8%)	4 (21.1%)	12 (46.2%)	13 (48.2%)	59 (56.7%)
Support with latching	19 (59.4%)	3 (15.8%)	8 (30.8%)	8 (29.6%)	38 (36.5%)
Support with expressing breastmilk	6 (18.8%)	2 (10.5%)	21 (80.8%)	12 (44.4%)	41 (39.4%)
Support for feeding with bottle/cup/paladai	3 (9.4%)	2 (10.5%)	20 (76.9%)	5 (18.5%)	30 (28.9%)
Other	2 (6.3%)	0	10 (38.5%)	0	12 (11.5%)
**OBSERVATION: Provider of support**	**N = 32**	**N = 19**	**N = 26**	**N = 27**	**N = 104**
Healthcare provider**	29 (90.6%)	7 (36.8%)	26 (100.0%)	27 (100.0%)	89 (85.6%)
Lactation consultant	2 (6.3%)	0	0	0	2 (1.9%)
Community health worker	1 (3.1%)	2 (10.5%)	0	0	3 (2.9%)
Family member	17 (53.1%)	13 (68.4%)	2 (7.7%)	0	32 (30.8%)
Friend	3 (9.4%)	0	0	1 (3.7%)	4 (3.9%)
Other	0	0	0	0	0

* Not able to distinguish between physical and verbal support in some cases

**Inclusive of doctor, nurse, and midwife

#### Breastfeeding competency

Feeding competency assessments showed significant improvement from baseline to facility discharge across all sites (**S2 Fig)**. The average PIBBS score at baseline was 11.9 (±4.3) and increased to 14.2 (±3.3) within 24 hours prior to discharge; 58.6% of infants were discharged with low feeding competency (PIBBS score ≤15). There were no significant differences in the mean discharge PIBBS scores by lactation support, sex, LBW type, size-for-gestational age, or GA.

### Discharge procedures

Based on national and facility policy, infants met certain criteria around feeding, stability, and weight to be discharged. Of the 140 infants assessed at discharge, 97.1% were fed only breastmilk (69.3% direct, 7.1% expressed only, 20.7% both direct and expressed), 2.7% fed formula only, and 0.7% fed a mix of formula, direct and expressed milk. Weight change between birth and discharge was minimal (**Table 5**). On average, infants lost 2.4% (± 5.8%) of their birthweight, varying by length of stay. Overall, 5.1% (7/138) lost >10% of their birthweight at the time of discharge. However, 18.8% of infants were discharged with weight below the site specified discharge criteria: 20.6% (7/34) of infants in India-Karnataka and 5.7% (2/35) with weights <1800g; 40.0% (16/40) in Tanzania had <2000g; (3.5%) (1/29) in Malawi <1500g. All infants were stable at the time of discharge.

**Table 5:** Change in weight (grams) from birth to discharge by length of stay for 148 moderately low birthweight infants in 12 facilities in India, Malawi, and Tanzania.

Length of stay (in days)	Birthweight (in grams)			Discharge weight (in grams)			Change in weight from birth to discharge (in grams)		Birth-weight regain prior to discharge (n (%))
	N	Mean (SD)	Median (IQR)	N*	Mean (SD)	Median (IQR)	Mean (SD)**	Mean percent (% (SD))	
0 to 2	47	2184 (201)	2200 (2070, 2345)	43	2120 (190)	2120 (1978, 2270)	-68 (66)	-3.1 (3.0)	5 (10.6)
3 to 4	40	2088 (232)	2110 (1890, 2265)	39	2019 (170)	2000 (1900, 2125)	-68 (128)	-2.8 (6.3)	10 (25.0)
5 to 7	38	2076 (245)	2118 (1925, 2280)	34	2032 (265)	2068 (1885,2260)	-45 (91)	-2.2 (4.3)	12 (31.6)
8 to 14	15	1992 (322)	2025 (1700, 2270)	14	1965 (394)	1948 (1580,2288)	-40 (174)	-2.2 (8.8)	6 (40.0)
15 plus	8	1786 (293)	1708 (1595, 1990)	8	1794 (250)	1850 (1578, 2015)	9 (208)	1.3 (11.9)	5 (62.5)
**Overall**	**148**	**2089 (255)**	**2145 (1924, 2280)**	**138**	**2035 (245)**	**2055 (1900, 2226)**	**-55 (115)**	**-2.4 (5.8)**	**38 (25.7)**

*In some instances, smaller denominators are noted for the subsequent analyses involving birth and discharge weights due to 10 (6.8%) missing discharge weights, which resulted from discharge during a weekend with no study staff on duty, infant death, or lack of documentation

** Change in weight was calculated among those infants with both birth and discharge weights; sample size is the same as those with a discharge weight

## Discussion

MLBW infants represent a heterogeneous group with different needs and implications for care and utilization of limited resources. Across the 12 facilities in 3 countries, we observed high adherence to EBF and relatively short lengths of stay (average 3 days), yet notable levels of mother-infant separation and gaps in universal and repeated lactation support or counseling [22–24].

Uptake and adherence to EBF was high, aligning with global recommendations and facility standard protocols [22, 25]. More than 80% of infants had early initiation of breastfeeding, which is higher than the global average (57.6%) [26–28]. Further, 97.1% of infants were discharged EBF, again higher than expected [29]. In our study, EBF was supported with utilization and promotion of breastmilk expression. Additional methods to aid with lactogenesis and support milk expression, including safe storage and increased availability of pumps in the facility, may be beneficial. Nearly 30% of infants were fed expressed breastmilk at discharge, which may be challenging to sustain at home given the need for clean water to wash breast pump materials to safely feed expressed breastmilk [30].

While uptake of EBF was excellent, health system constraints (e.g., bed capacity, human resources, limited specialized LBW training) likely led to misalignment of practice and policy and limited optimization of in-facility care, demonstrated by <60% receiving 2+ lactation support sessions, >66% of dyads experiencing maternal-newborn separation, and discharge of 18.8% of infants below minimum weight thresholds. Nurse-to-patient ratio guidelines from medical and nursing societies range from 1:3 for the lowest acuity care (level 1) to 1:1 for the highest acuity care (level 5) [31–33]. Only three facilities met nurse-to-patient guidelines for the lowest acuity care at level 1 facilities (1:3); some facilities had ratios as low as 1:35 infants. Increasing health worker capacity has been identified as a core standard for improving quality of maternal and newborn care in health facilities [34], meeting global recommendations [22], and improving neonatal outcomes [35, 36].

Lack of universal and hands-on provision of lactation support and counseling was possibly driven by the high levels of mother-infant separation, staff shortages, and lack of specialized training on LBW lactation support among providers, which are well-documented barriers to support of EBF [37–41]. Unresolved breastfeeding problems, highlighted by poor feeding competency scores, can lead to growth faltering, reduced duration of EBF, subsequent hospitalizations, and mortality [42, 43]. Group counseling and support in the facility and in-home peer-counseling interventions have shown improved EBF initiation and duration [41]; these strategies could be adapted specifically for LBW infants to improve in-facility support.

Further, breastmilk alone may be insufficient to meet the nutritional needs of MLBW infants [42, 44–46]; most facilities did not have alternatives readily available. Only one facility had access to parenteral nutrition; one site supplemented infants with micronutrients; protein supplementation and fortification of human milk were not used at any site. When appropriate and safely administered, DHM and formula can provide a bridge to breastfeeding [46–48]. However, a human milk bank was only at one facility and formula availability was limited; a potential barrier of formula use was the cost to families. Finally, of note, we found a high prevalence of discharge among infants still demonstrating postnatal weight loss (Table 5) and/or discharge prior to reaching minimum weight benchmarks as stipulated by facility discharge policies. Weight loss among LBW and vulnerable infants is of critical importance and requires additional in-facility care measures including daily monitoring of anthropometrics, clinical documentation, and plans for continuity of care for post-discharge monitoring to ensure LBW infants do not become further growth-restricted.

Our study addresses key knowledge gaps as the majority of published research focuses on very LBW/preterm infants in high-income settings despite the predominance and risk of MLBW infants [7]. We present detailed data from three diverse countries and twelve hospitals. Multiple data collection methods, including facility needs assessment, direct observations, and maternal reports, allowed for triangulation of information.

We do have some notable limitations. We focused on MLBW infants, which preselected a preterm-AGA population that is less mature than a preterm-SGA group, (i.e., most early preterm-SGA infants have birthweights <1500g and were therefore ineligible). In addition, our study population included infants born at secondary and tertiary facilities and thus cannot be generalized to infants born at lower-level facilities or at home. The study sample size allowed for a deep understanding of the feeding experiences and care of LBW infants and resulted in a rich descriptive data at each site. However, this sample does not represent all LBW infants and, since this was intended largely as a descriptive paper, our modeling analyses are limited. Statistically significant findings should be interpreted cautiously as we could not control for potential confounders, and observed associations may be spurious. Further studies need to confirm our findings. Additionally, GA source differed by site which could cause LBW type misclassification; however, we use prioritization criteria for choosing the source of GA, namely ultrasound at first trimester when available over other sources followed by LMP in medical charts. Finally, observation by study staff may have altered participant behaviors; however, we had multiple observations over time and had specific research nurses gather information rather than clinical staff.

## Conclusion

With increases in facility-based childbirth, critical opportunities exist to set up MLBW infants, their mothers, and families to thrive post-discharge. Given the renewed investment and recent guidelines in the care of small and sick newborns [49, 50], the global community must identify gaps and at-risk groups, and modify strategies to support the adherence to policies like BFHI, zero-separation, and minimum discharge weights. Our study fills a critical gap in foundational knowledge on the health system inputs and current feeding and care practices for MLBW infants in resource-constrained settings. This is a group for whom evidence is limited, but the potential to survive and thrive is high [4, 51, 52]. Our findings highlight areas of focus to improve the quality of care delivered to MLBW infants including health systems improvements to increase human capacity, staffing and training, to improve breastfeeding alternatives availability, and to support adherence to discharge policies, which would improve mother-infant experience of care by reducing mother-infant separation, initiating breastfeeding early, and providing lactation support and counseling specialized for MLBW.

## Supporting information

S1 Fig Observed infant feeding patterns from birth to discharge among infants discharged after 7 days of life across 12 facilities in India, Malawi, and Tanzania. (TIF)

S2 Fig Infant feeding competency scores assessed by PIBBS at baseline and 24 hours prior to facility discharge in a cohort of moderately LBW across 12 facilities in India, Malawi, and Tanzania. (TIF)

S1 File PLOS global public health inclusivity questionnaire. (DOCX)
