# Supplementary figures and images for: Facility-based care for moderately low birthweight infants in India, Malawi, and Tanzania
Source: PLOS Glob Public Health. 2023 Apr 19;3(4):e0001789. doi: 10.1371/journal.pgph.0001789 (PMC10115266; doi:10.1371/journal.pgph.0001789)

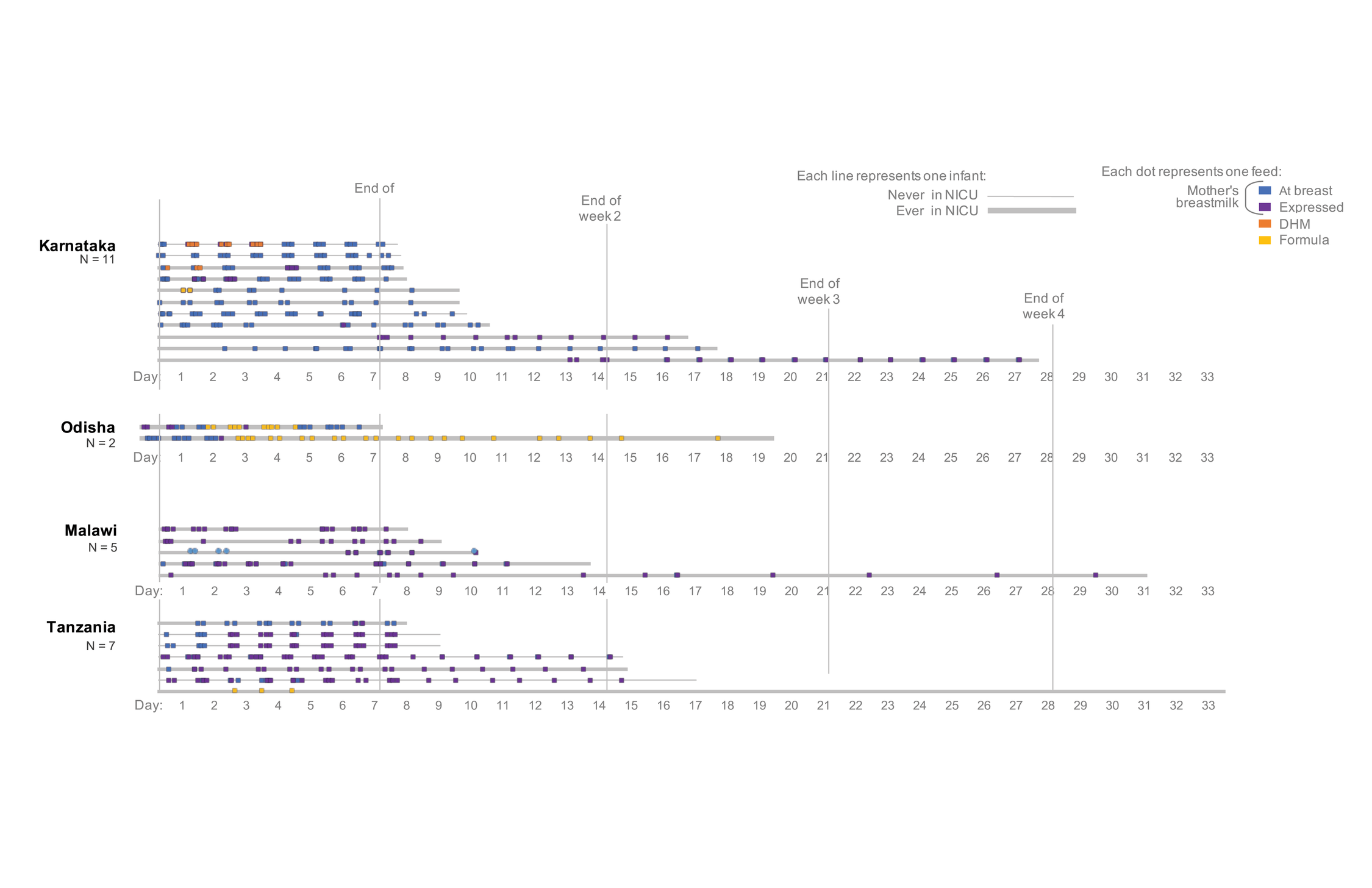

Supplement: S1 Fig — (TIF) [file pgph.0001789.s001.tif]

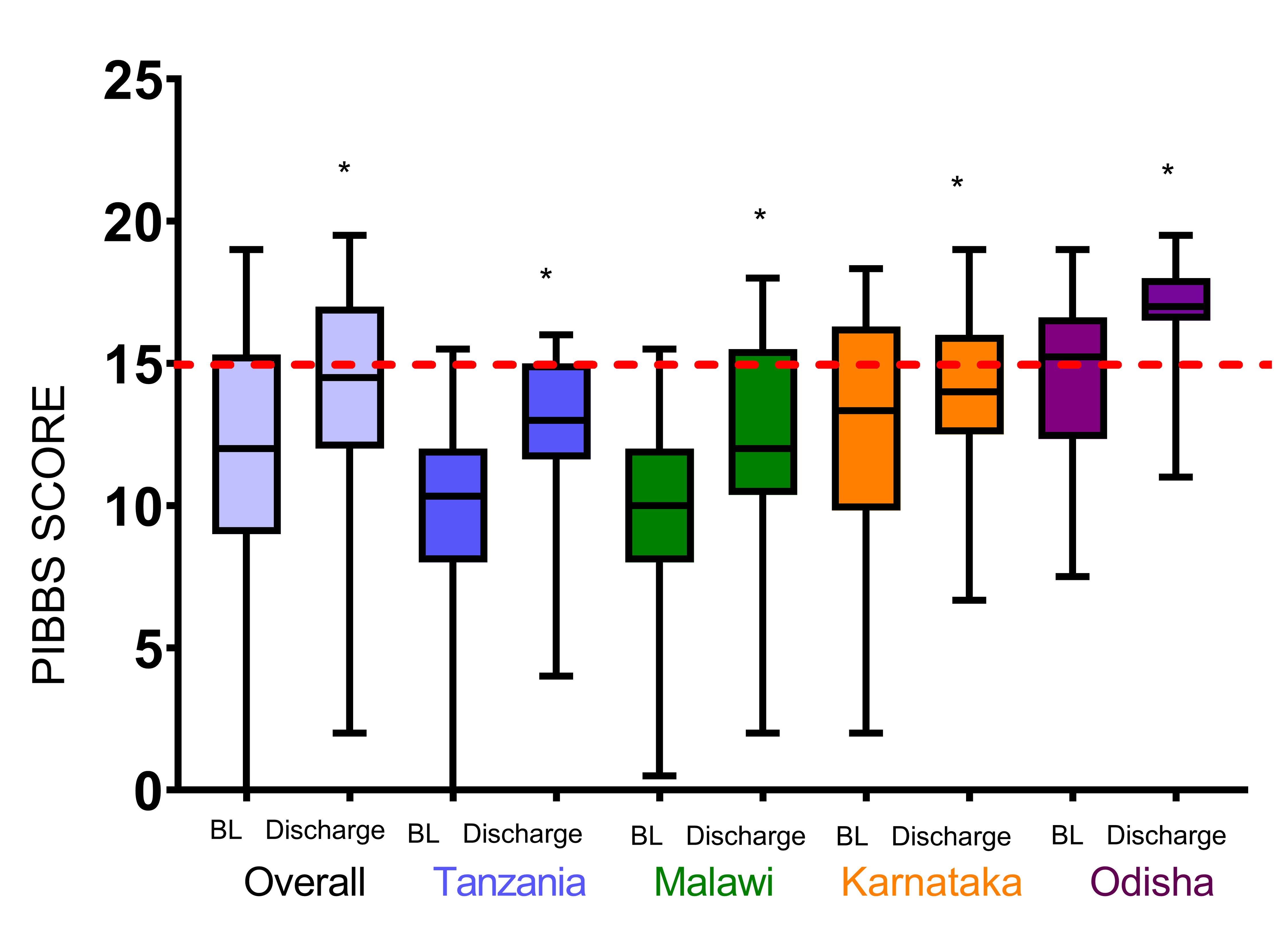

Supplement: S2 Fig — (TIF) [file pgph.0001789.s002.tif]
