# Supplementary material for: Facility-based care for moderately low birthweight infants in India, Malawi, and Tanzania
Source: PLOS Glob Public Health. 2023 Apr 19;3(4):e0001789. doi: 10.1371/journal.pgph.0001789 (PMC10115266; doi:10.1371/journal.pgph.0001789)
Supplement: S1 File — (DOCX) [file pgph.0001789.s003.docx]

Inclusivity in global research

PLOS’ policy on inclusivity in global research aims to improve transparency in the reporting of research performed outside of researchers’ own country or community and ensures that PLOS publications reporting global research adhere to high standards for research ethics and authorship. Authors of relevant research articles may be asked to complete the questionnaire below, which outlines ethical, cultural, and scientific considerations specific to inclusivity in global research. This questionnaire may be requested when researchers have travelled to a different country to conduct research, if research uses samples collected in another country, research with Indigenous populations or their lands, or if research is on cultural artefacts. Researchers travelling to another country solely to use laboratory equipment will not normally be required to complete the questionnaire. However, the questionnaire can be requested at the journal’s discretion for any submission – if you have been requested to complete this questionnaire by the PLOS journal you submitted to, please do so.

Please complete the questionnaire below and include this as a Supporting Information file with your manuscript. Note that if your paper is accepted for publication, this checklist will be published with your article in the supporting information files. Please ensure that you reference the checklist in the main body of your manuscript. We suggest adding a subsection ‘Inclusivity in global research’ to your Methods section and adding the following sentence: “Additional information regarding the ethical, cultural, and scientific considerations specific to inclusivity in global research is included in the Supporting Information (SX Checklist)”

The questions have been designed to be applicable to a wide range of study types, and there are subsections for both human subjects research and non-human subjects research. If any of the questions are not relevant to your research please mark them as “N/A” as appropriate.

**Ethical considerations, permits and authorship**

*This section is applicable to all research types.*

Provide details as to who granted permissions and/or consent for the study to take place in the Methods section of your manuscript. This should include the names of **all** ethics boards, governmental organizations, community leaders or other bodies that provided approval for the study. If individuals provided approval refer to these people by their role or title but do not list their name(s).

Reported on page number: 8, 32-33

If there were any deviations from the study protocol after approval was obtained please provide details of these changes in the Methods section of your manuscript.
Did this study involve local collaborators that are residents of the country where the research was conducted or members of the community studied? If you do not have any authors from said communities, please provide an explanation for this below.

This study does not have any deviations from the study protocol. This study does involve collaborators who are residents of the countries in which the study was conducted and co-developed the study design. We have tried to be as inclusive as possible in co-authorship reflecting the work completed in the study design, data collection, data analysis and interpretation and writing.

Everyone listed as an author should meet PLOS’ criteria for authorship and all individuals who meet these criteria should be included in the author byline, rather than the acknowledgements. Authorship criteria is based on the International Committee of Medical Journal Editors (ICMJE) Uniform Requirements for Manuscripts Submitted to Biomedical Journals - for further information please see here: <https://journals.plos.org/plosone/s/authorship>.

All authors meet the ICJME criteria and PLoS Global Public Health authorship. We have also included the following reflexivity statement in the additional information of the manuscript:

The research study was co-developed by a consortium of 14 organizations and more than 50 team members in India, Malawi, Tanzania, and the United States. Research objectives were determined based on priority areas and evidence gaps identified through review of the existing literature and by site partners and subject matter experts. A publications committee, consisting of study PIs from India, Malawi, Tanzania and the United States, developed an inclusive and representative writing process, determination of authorship, and strategy to provide writing opportunities for early career team members. The committee utilized the ICJME criteria and defined clear principles of diversity, equity and inclusion. With more than 42 authors on this paper, including early career team members in all organizations, data collectors, researchers, and clinicians, we believe that we have attempted to be inclusive. Although the first and last authors are not from low- and middle-income countries (LMIC), this authorship order was proposed and agreed on by the publication committee. At every stage of the LIFE study, preliminary and final findings have been shared with LMIC partners and have been refined and iterated based on their feedback and contextual interpretations. In addition, all findings were shared with all members of the consortium in order to maximize their dissemination. Our funder, the Bill and Melinda Gates Foundation, strongly encourages and financially supports the dissemination of findings in open access journals; this is a priority of our consortium and a reason we have submitted to PLoS Global Health*.*

**Human subjects research (e.g. health research, medical research, cross-cultural psychology)**

Did you obtain written informed consent from a representative of the local community or region before the research took place? How did you establish who speaks for the community? Details of written informed consent obtained from study participants should be reported separately in the Methods section of your manuscript.

How did members of the local community provide input on the aims of the research investigation, its methodology, and its anticipated outcome(s)?

As noted above, this consortium of 14 organizations, including scientists and researchers from the community, designed the research study and aims in a collaborative process during proposal development.

When engaging with the local community, how did you ensure that the informed consent documents and other materials could be understood by local stakeholders?

All consent materials were translated and back translated into local languages as appropriate. In addition to the written material, study team members who conducted enrollment talked through the consent forms and study procedures prior enrollment. Consent forms were piloted in the early phases of the project with women/families.

Will the findings of the research be made available in an understandable format to stakeholders in the community where the study was conducted (e.g. via a presentation, summary report, copies of publications, etc.)? Please provide details of how this will be achieved.

Yes, the results have been shared with stakeholders via a PPT presentation and/or a short executive summary/brief capturing the key results. Country partners have conducted dissemination via a national research conference or stakeholder meetings.

**Non-human subjects research using specimens/ animals collected as part of the study, or those housed in archival collections. Examples include archaeology, paleontology, botany and zoology.**

Did the permission you obtained from a local authority to perform the study include an agreement on access to outputs and benefit sharing? This may include procedures to enable fair distribution of the benefits and resources arising from the research performed. Please include any details of Prior Informed Consent and Benefit Sharing Agreements obtained. These may be required by field-specific regulations, for example the Convention on Biological Diversity (CBD) and the associated Nagoya Protocol.

N/A this was human subjects research

If the material used in your study was imported, please A) provide the year it was imported and B) indicate whether permits were obtained to import/export the materials used, C) provide details of any permits obtained. If this information is not available, please indicate this.

N/A

If you used archival specimens, please state how the material used in your study was acquired by the institute it is held in and provide details of any permits obtained for the original excavations/ sample collection. If this information is not available, please indicate this.

N/A

How was the potential cultural significance of the materials collected in your study to local communities considered in your research design? Were Indigenous peoples and/or local researchers and institutions involved with archaeological excavations / collection of specimens? If so, please provide a description of their involvement.

N/A

If your manuscript includes photographs of human remains please indicate whether authors obtained permission from descendants or affiliated cultural communities to do so.

N/A
